# Regulation of the Innate Immune Response during the Human Papillomavirus Life Cycle

**DOI:** 10.3390/v14081797

**Published:** 2022-08-17

**Authors:** Cary A. Moody

**Affiliations:** 1 Lineberger Comprehensive Cancer Center, University of North Carolina at Chapel Hill, Chapel Hill, NC 27599, USA; camoody@med.unc.edu; 2 Department of Microbiology and Immunology, University of North Carolina at Chapel Hill, Chapel Hill, NC 27599, USA

**Keywords:** HPV, IFN, innate immunity, life cycle

## Abstract

High-risk human papillomaviruses (HR HPVs) are associated with multiple human cancers and comprise 5% of the human cancer burden. Although most infections are transient, persistent infections are a major risk factor for cancer development. The life cycle of HPV is intimately linked to epithelial differentiation. HPVs establish infection at a low copy number in the proliferating basal keratinocytes of the stratified epithelium. In contrast, the productive phase of the viral life cycle is activated upon epithelial differentiation, resulting in viral genome amplification, high levels of late gene expression, and the assembly of virions that are shed from the epithelial surface. Avoiding activation of an innate immune response during the course of infection plays a key role in promoting viral persistence as well as completion of the viral life cycle in differentiating epithelial cells. This review highlights the recent advances in our understanding of how HPVs manipulate the host cell environment, often in a type-specific manner, to suppress activation of an innate immune response to establish conditions supportive of viral replication.

## 1. Introduction

Human papillomaviruses (HPV) are small, non-enveloped, double-stranded DNA viruses that exhibit a strict tropism for epithelial cells. Over 400 types of HPVs have been identified, which are classified into five different genera (alpha, beta, gamma, mu, and nu) and target the stratified epithelium at different body sites [1]. HPVs cause a wide variety of type-specific pathologies, ranging from hyperproliferative lesions to clinically inapparent or asymptomatic infections that can progress to high-grade neoplasms and cancers in certain individuals [2]. The alpha HPV types that infect the mucosal epithelium are classified as low-risk or high-risk based on their association with cancer. Approximately 15 HPV types (e.g., HPV16, 18, 31, 45) are considered high-risk and are associated with 5% of human cancers. High-risk HPVs (HR HPVs) are the causative agents of cervical cancer, with 99% of cervical cancers containing HR HPV DNA [3]. HPV16 and HPV18 are associated with 50% and 20% of cervical cancers, respectively. HR HPV infections are also associated with the development of other genital malignances as well as an increasing number of head and neck cancers, which are predominantly associated with HPV16 [4,5]. HPV-induced carcinogenesis requires the activities of the E6 and E7 oncoproteins. The transforming activities of E6 and E7 are mainly carried out through protein–protein interactions that are important for establishing a replication-competent environment in the stratified epithelium [6,7,8,9]. Currently, there are three FDA-approved prophylactic HPV vaccines that are estimated to have the potential to prevent 70–90% of HPV-associated cancers [10]. However, these vaccines are not therapeutic against pre-existing infections and lesions.

Although the lifetime risk of developing a genital HPV infection is greater than 80%, most of these infections are transient. On average, however, clearance of viral infections by the immune response can take 1–2 years, indicating that HPVs have evolved mechanisms to escape immune surveillance [11]. Additionally, viral persistence is a key requirement for the development of cancer, with cervical cancer occurring decades after initial infection. The importance of the immune response in clearing HPV infections is evidenced by the increased risk of HPV infection and the development of cervical cancer in HIV-infected women and other immunosuppressed patients [12,13]. Although E6- and E7-induced DNA damage and genomic instability are important for cancer development [14], the ability to avoid an immune response is necessary to promote viral persistence as well as for completion of the viral life cycle [15]. HPV accomplishes this through the organization of the viral life cycle as well as the ability of viral proteins to actively target components of innate immune signaling pathways. These mechanisms are discussed below.

## 2. The HPV Genome

**Figure 1:**
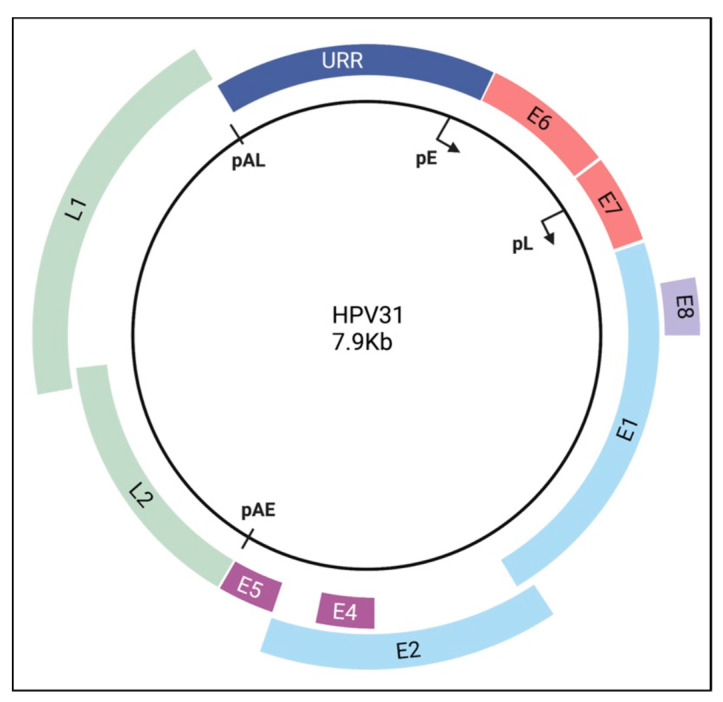
Genomic organization of high-risk HPV31. The open reading frames are designated by the color blocks. The early promoter (pE) is located upstream of the E6 ORF (p97 for HPV16, HPV31; p105 for HPV18), and the late promoter (pL) is located in the E7 ORF (p742 for HPV31, p811 for HPV18, p670 for HPV16). Expression of E8^E2C is driven by a promoter in the E1 ORF (not shown). The early polyadenylation site (pAE) is located at the end of the E5 ORF, and the late polyadenylation site (pAL) is located at the end of the L1 ORF. The upstream regulatory region (URR) is an untranslated region that contains the keratinocyte enhancer, the origin of replication, as well as binding sites for E1 and E2 and various transcription factors. Created with BioRender.com (accessed on 18 January 2022).

All HPV genomes are approximately 8 kb and exist in infected cells as extrachromosomal elements (episomes). The viral DNA is histone-associated in the virion as well as in infected cells and is subject to epigenetic regulation through histone post-translational modifications as well as DNA methylation [16]. HPVs share a common genomic organization with six to eight open reading frames (ORF), consisting of an early (E) region, a late (L) region, and a regulatory region called the long control region (LCR) (e.g., HPV16, 18) or the upstream regulatory region (URR) (e.g., HPV31) (Figure 1) [14]. There are two main promoters that are active at different phases of the viral life cycle. The early genes (E1, E2, E6, E7, E8) are expressed at low levels prior to epithelial differentiation from a promoter that is located in the URR/LCR. E1 is an ATP-dependent helicase that is required for viral replication along with E2 [17]. E2 also regulates viral gene expression from the early promoter and facilitates viral genome retention upon cell division by tethering viral genomes to host mitotic chromosomes [18]. E6 and E7 are the viral oncoproteins and support viral replication by deregulating cell cycle control, delaying differentiation, and antagonizing innate immune pathways [19,20]. Some HPV types express E8^E2C, which is a fusion of E8 and the C-terminal half of the E2 ORF. E8^E2C is transcribed from a promoter in the E1 ORF and functions to limit viral replication and transcription in undifferentiated and differentiated cells [21]. E4 and E5 are contained on early transcripts but are only highly expressed upon epithelial differentiation, which triggers activation of the late promoter located in the E7 ORF. E4 is expressed as a spliced transcript that fuses the first five amino acids of the E1 ORF with E4 (E1^E4). E1^E4 is required for productive replication of high-risk HPV16, 18 and 31 [22]. E5 has been shown to contribute to productive replication of HPV31 and HPV18 [23,24]. Activation of the late promoter also results in high level expression of E1 and E2 as well as the immunogenic capsid proteins L1 and L2 [25].

## 3. The HPV Life Cycle

**Figure 2:**
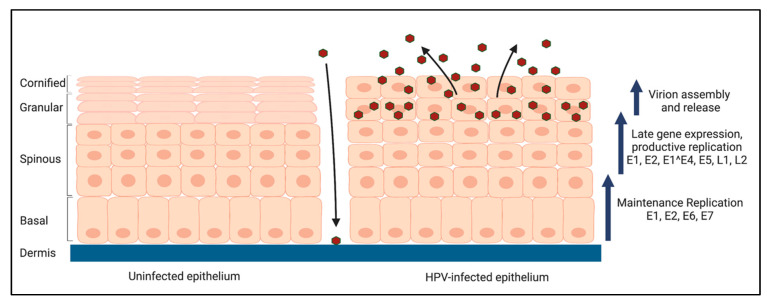
Overview of the HPV life cycle. HPVs infect the basal cells of the stratified epithelium through a microwound and transiently amplify to 50–100 copies per cell. Episomal copies are thought to be maintained at a low copy number in the undifferentiated, basal epithelial cells by replicating along with cellular DNA. Differentiation triggers the productive phase of the life cycle, resulting in high levels of late genes being expressed, including E1 and E2, that facilitate amplification of viral genomes to 100–1000 s of copies per cell. E1^E4 and E5 are also highly expressed upon differentiation and contribute to providing a replication-competent environment. E6 and E7 promote re-entry of differentiating cells back into the cell cycle to provide cellular factors required for viral replication. The expression of L1 and L2 results in the assembly of new virions that are released from the uppermost layers of the stratified epithelium. Created with BioRender.com (accessed on 10 August 2022).

The HPV life cycle is linked to epithelial differentiation [14]. HPVs establish a persistent infection in the mucosal and cutaneous epithelium by infecting the proliferating, basal cells of the stratified epithelium that are exposed through a microwound [2,26]. Nuclear envelope breakdown upon cell division allows viral entry into the nucleus [27,28]. The HPV genome is then rapidly amplified to 50–100 episomal copies per cell and subsequently stably maintained in these undifferentiated cells [29]. Epithelial differentiation triggers the productive phase of the viral life cycle, resulting in viral genome amplification to 100–1000 s of copies per cell, high levels of late genes transcribed from the late promoter, including the immunogenic capsid proteins L1 and L2, which facilitate virion assembly [14]. Viral particles are then released from the uppermost layers of the stratified epithelium (Figure 2).

The limited coding capacity of the HPV genome renders the virus reliant on cellular factors for viral replication. However, epithelial differentiation normally results in an exit from the cell cycle. To provide an environment conducive to productive replication, HPV has evolved mechanisms to push differentiating cells back into the cell cycle, largely through E6 and E7′s ability to deregulate cell cycle control, including the degradation of the p53 and pRb tumor suppressors, respectively [25]. Productive replication occurs post-cellular DNA synthesis in a G2-arrested environment and relies on activation of DNA damage response pathways [30,31]. By restricting productive replication, late gene expression, and virion assembly to the uppermost layers of the stratified epithelium, HPV is able to avoid immune surveillance in the basal layers. However, HPV must also employ strategies to avoid activating an innate antiviral response during the productive phase of the viral life cycle.

## 4. Innate Antiviral Signaling Pathways

**Figure 3:**
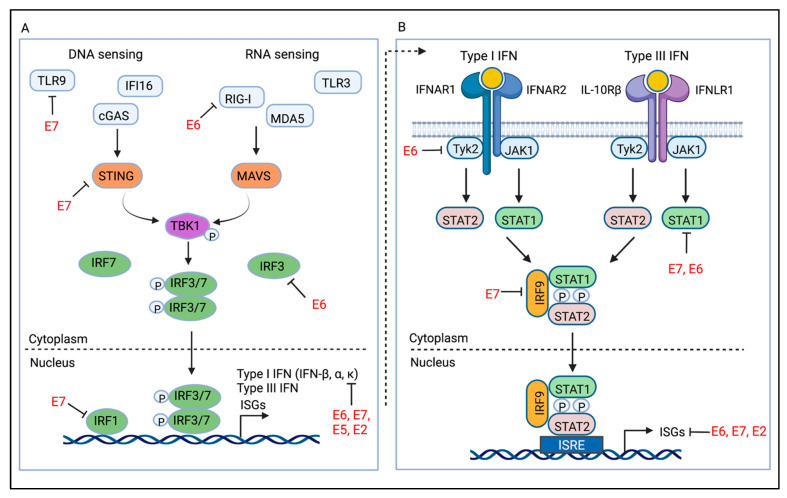
Overview of pattern recognition receptor (PRR) and IFN signaling and interference by HPV early proteins. (**A**) Recognition of viral nucleic acids by DNA sensors (TLR9, cGAS, IFI16) and RNA sensors (TLR3, RIG-I, MDA5) leads to the induction of Type I and Type III IFNs as well as a subset of ISGs (e.g., ISG56, ISG54). (**B**) Secreted IFNs can act in an autocrine or paracrine manner to induce JAK/STAT signaling and ISG expression. Type I IFNs bind to a heterodimeric receptor comprisingIFNAR1 and IFNAR2 subunits, whereas Type III IFNs bind to a heterodimeric receptor comprising IFNLR1 and IL10Rβ subunits. Receptor dimerization activates Tyk2 and JAK1, which phosphorylate STAT1 and STAT2. Phosphorylated STAT1 and STAT2 heterodimers interact with IRF9 to form the ISGF3 transcription factor complex, which translocates to the nucleus and binds to IFN-sensitive response elements (ISREs) to drive expression of ISGs. Type I IFNs can also signal through STAT1 homodimers, and Type III IFNs can signal though JAK2. The HPV early proteins target multiple aspect of innate immune signaling to subvert the antiviral response. Created with BioRender.com (accessed on 18 July 2022).

The interferon (IFN)-mediated response serves as the first line of defense against viral infection. The IFN family is divided into three types; Type I (e.g., IFN-α, β, κ), Type II (e.g., IFN-γ), and Type III (IFN-λ1, 2, 3, 4). The Type I and Type III IFN responses are initiated for the most part by the recognition of pathogen associated molecular patterns (PAMPS), mainly viral nucleic acids, by multiple pattern recognition receptors (PRR) (Figure 3A) [32,33]. These PRRs include toll-like receptors (TLRs), RIG-I like receptors (RLRs) (e.g., RIG-I (retinoic acid–inducible gene-I), MDA5 (melanoma differentiation-associated gene 5), LGP2), cGAS (cyclic guanosine monophosphate–adenosine monophosphate synthase), and IFI16 (interferon gamma inducible protein 16). TLRs are transmembrane proteins that span the plasma or endosomal membrane and signal through different sets of adaptor proteins [34]. The endosomal TLRs recognize nucleic acids, which can be single-stranded RNA (ssRNA) (e.g., TLR7, TLR8), double-stranded RNA (dsRNA) (e.g., TLR3), or DNA (e.g., TLR9). RIG-I and MDA5 recognize distinct forms of dsRNA in the cytosol but use the common adaptor MAVS (mitochondrial antiviral-signaling protein) for signaling [35,36,37,38,39,40,41,42,43]. LGP2 is thought to regulate the activity of RIG-I and MDA5 through its ability to bind to RNA [44]. cGAS is a DNA sensor that is located in the cytoplasm as well as the nucleus, where it is tethered and kept inactive by chromatin [45,46]. When bound to double-stranded DNA (dsDNA), cGAS catalyzes adenosine 5′-triphosphate and guanosine 5′-triphosphate into cyclic GMP-AMP (2′3′cGAMP) [47,48]. cGAMP binds to and activates the adaptor STING (stimulator of interferon genes), which localizes to the endoplasmic reticulum membrane [49,50]. IFI16 is a DNA sensor that resides in the nucleus as well as cytoplasm and can also use STING as an adapter [51,52,53].

Despite recognizing distinct PAMPs, the cGAS-STING and RLR-MAVS pathways converge on activation of TANK-binding kinase 1 (TBK1), which phosphorylates and activates the transcription factors IRF-3/IRF-7 to induce Type I and Type III IFN expression (Figure 3A) [54]. IFNs are then secreted from the cell where they can bind in an autocrine or paracrine manner to their cognate receptors [55]. Receptor/IFN binding stimulates the JAK (Janus kinase)/STAT (signal transducer and activator of transcription) signaling cascade, resulting in the expression of hundreds of IFN stimulated genes (ISGs) that promote pathogen clearance (Figure 3B) [56].

Keratinocytes, the target cells of HPV, are immune sentinels that express several PRRs and can respond to PAMPs to mediate an immune response [57]. Genome-wide transcriptome studies have demonstrated that undifferentiated keratinocytes maintaining HPV18, HPV16, or HPV31 genomes episomally, or expressing the E6 and E7 oncoproteins, exhibit reduced levels of ISGs compared to uninfected keratinocytes, indicating that HPV interferes with components of the innate immune response [58,59,60]. Indeed, several mechanisms have been identified by which HPV deregulates the innate immune response, including interfering with IFN production as well as blocking ISG expression. Several ISGs have been shown to restrict HPV replication, including STAT1, which is an essential component of JAK/STAT signaling leading to ISG expression, and IFIT1, which has been reported to bind to and inhibit the HPV11 and 18 E1 helicase [61,62,63]. Furthermore, long-term treatment of high-risk HPV31 or HPV16-infected keratinocytes with recombinant IFN-β leads to the loss of episomes and outgrowth of cells containing integrated genomes [64,65]. Additionally, the emergence of cells containing integrated HPV16 genomes after long-term culture is associated with an antiviral response and episomal loss [66]. Inactivating the IFN response is therefore key in all phases of the viral life cycle to promote viral replication and episomal persistence.

Multiple approaches have been used to investigate the mechanisms by which HPVs interfere with activation of an innate immune response. Infection of keratinocytes with HPV pseudovirions (L1/L2 encasing a reporter plasmid), quasivirions (L1/L2 encasing the HPV genome), or virions harvested from HPV positive organotypic raft cultures have been used to study early events in HPV infection [67,68,69]; HPV positive keratinocytes derived from CIN1 (cervical intraepithelial neoplasia grade 1) lesions (e.g., CIN612 9E- HPV31 positive; W12E- HPV16 positive) as well as keratinocytes transfected with wild-type (WT) or mutant viral genomes have been used to study the maintenance as well as productive phases of the viral life cycle [70,71,72]. Heterologous expression systems have been used to study the impact of the HPV early genes on the expression and function of components of the innate immune response. These various approaches have revealed that HPVs target multiple nodes in the antiviral signaling response, often in a type-specific manner, to interfere with nucleic acid sensing, IFN production and signaling, and ISG expression.

## 5. Activation of the Innate Immune Response upon Initial HPV Infection

Innate surveillance pathways serve as a major obstacle to viral infection. Indeed, many DNA viruses have been shown to activate cGAS/STING during entry, trafficking and uncoating, including herpesviruses (e.g., herpes simplex virus type 1 (HSV-1), Kaposi’s sarcoma associated herpesvirus (KSHV)), adenoviruses, and poxviruses [73,74,75,76]. HPVs use the minor capsid protein L2 to transport viral DNA within vesicular membranes to the nucleus [77]. Uhlorn et al. found that perturbation of the vesicular membranes leads to sensing of HPV16 pseudovirus infection by cGAS/STING, indicating that vesicular membrane trafficking effectively shields viral DNA from cytosolic PRRs during nuclear transit [78]. The endocytic pathway of HPV entry could expose viral DNA to endosomal TRL9, which recognizes unmethylated CpG motifs in dsDNA viral genomes [79,80]. Interestingly, however, many papillomaviruses exhibit reduced CpG content, including cancer-associated Alphapapillomaviruses, which may prevent detection by TLR9 [81,82,83]. Whether HPV genomes are hypomethylated in the virion is currently unknown. However, Hasan et al. showed that keratinocyte depletion of TLR9 using a small hairpin RNA (shRNA) results in a higher viral copy number following HPV16 quasivirus infection [84], suggesting that virion DNA is hypomethylated.

Upon entry into the nucleus, PML (promyelocytic leukemia) proteins associate with and assemble around HPV genomes [85]. PML protein is a component of nuclear bodies (NBs) that are antiviral in nature and typically targeted for disassembly/reorganization by DNA viruses [86]. However, HPV requires PML protein for efficient establishment of infection [87,88,89,90]. A recent study from Martin Sapp’s group suggests that PML protects viral genomes from innate and intrinsic sensors, allowing for retention of viral DNA in the nucleus and transcription [85]. They show that the recruitment of Sp100, an ISG that is a transcriptional repressor and component of NBs [91], to viral genomes is delayed compared to PML [85], likely allowing an initial burst of viral transcription to promote infection. Indeed, as discussed below, Sp100 has been shown to restrict early events during the initial stages of HPV18 infection [87]. Avoiding activation of these sensing pathways upon viral entry is likely critical to the establishment of infection and viral persistence.

## 6. Interference with IFN Induction

The establishment of infection allows production of HPV early proteins that can directly block proximal viral nucleic acid detection machinery of the cGAS-STING and RLR-MAVs pathways as well as disrupt components of antiviral signaling that are shared among DNA and RNA virus sensors. HPV interferes with multiple steps in the pathway to block cell intrinsic and extrinsic responses. The constitutive reduction in ISGs can be attributed at least in part to HPVs ability to suppress IFN production.

### 6.1. Targeting Nucleic Acid PRR-Adaptor Signaling

#### 6.1.1. DNA Sensors

The HPV18 and HPV16 E7 proteins suppress STING-dependent IFN responses through distinct mechanisms. HPV18 E7 binds to STING to antagonize its function [92]. In contrast, HPV16 E7 targets STING for degradation via autophagy by hijacking the PRR component NLRX1 [93]. In the context of the HPV genome, normal immortalized keratinocytes (NIKS) containing episomal HPV18 genomes exhibit reduced expression of DNA sensors, including cGAS and IFI16 as well as the adaptor STING, and respond poorly to exogenous DNA ligands [94]. STING levels are also decreased in HPV positive normal and low grade squamous intraepithelial lesions compared to HPV negative control lesions, indicating that targeting STING early is one mechanism to keep antiviral signaling in check [95]. HPV16 and HPV18 E7 have been reported to alter STING and cGAS levels through epigenetic repression mediated by the SUV39H1 methyltransferase [96]. HPV16 E7 has also been reported to epigenetically repress the levels of TLR9 [84], and reduced levels of TLR9 are observed in HPV16 positive keratinocyte lines and cervical cancers [97,98], suggesting that TLR9 signaling may impact HPV infection. Overexpression of the HPV18 and HPV16 E2 proteins is also sufficient to downregulate STING mRNA levels along with the several other innate immune genes [95,99]. The cGAS-STING pathway plays a critical role in the response to damaged DNA that is mislocalized to the cytoplasm as well as DNA within micronuclei [100,101]. HR HPVs induce DNA damage and require activation of DNA damage response pathways for viral replication [14,25]. The ability to interfere with PRR signaling likely allows HPV to replicate and maintain a high copy number despite the presence of DNA damage [102,103]. Additionally, the assembly of HPV DNA into nucleosomes at all stages of the life cycle likely precludes its recognition by cGAS/STING in the nucleus [104,105,106].

#### 6.1.2. RNA Sensors

The dsRNA sensors RIG-I and MDA5 are also targets of HPV early proteins. Through in vitro studies, Chiang et al. demonstrated that the HPV16 E6 protein inactivates two upstream activators of RIG-I, TRIM25 and USP15, and blocks the RIG-I-induced IFN response to Sendai virus infection [107]. E6 proteins from low- and high-risk HPV types can bind to TRIM25, suggesting this mechanism of RIG-I inhibition may be conserved among HPV types [107]. Using HPV16 virions harvested from organotypic raft cultures, Chiang et al. also showed that a RIG-I-dependent IFN response is detected 48 h post-infection of keratinocytes [107]. Although RIG-I and MDA5 are best characterized for restriction of RNA viruses, they have also been shown to initiate innate immune responses to several DNA viruses, including Adenovirus, KSHV, Epstein–Barr virus (EBV), and HSV-1 [108,109,110,111]. RNA polymerase III plays a role in the innate immune response to DNA viruses by transcribing cytosolic AT-rich viral DNA into short tri-phosphorylated non-coding RNAs that are recognized by RIG-I [110,111,112,113]. RIG-I sensing of DNA viruses can also occur through recognition of viral RNAs as well as misprocessed host RNAs [109,114]. The in vivo RNAs, whether host or viral, that are detected by RIG-I in response to HPV16 infection were not determined, nor was the effect of RIG-I-induced signaling on early events in viral infection [107]. However, as discussed below, our recent studies demonstrate that HPV’s ability to interfere with RLR-MAVS signaling is instrumental to suppress an IFN response during the productive phase of the viral life cycle [115].

### 6.2. IRF Activation

PRR-adaptor signaling induces IFN production through the activation of IRFs. Type I and Type III IFNs are induced by the activation of IRF3 and/or IRF7 through the activity of TBK1 [54]. Activated IRF3 and IRF7 then translocate to the nucleus to induce expression of IFN. Additionally, IFN-λ1 can be induced by IRF1 [116]. In addition to affecting the function and levels of PRRs, HPV also targets IRFs to block IFN production. HPV16 E6, but not HPV18 E6, binds to and interferes with the activity of IRF3 [117,118], whereas HPV16 E7 binds to and inhibits the activity of IRF1 [119]. Although these interactions may contribute to HPVs ability to evade activation of an innate immune response, the biological significance of these interactions in the context of HPV infection has not been examined.

### 6.3. Negative Regulation of IFN-κ Expression

The regulation of IFN-κ plays a critical role in HPV’s ability to dampen an innate immune response. IFN-κ is a Type I IFN that is constitutively expressed in keratinocytes and can drive basal levels of ISG expression [120]. In contrast to IFN-α/β and Type III IFN-λ, IFN-κ is minimally induced by PRR signaling and acts primarily in an autocrine manner to stimulate JAK/STAT signaling and ISG production [120,121]. Several studies have shown that IFN-κ expression is suppressed in keratinocytes containing high-risk HPV18, 16, or 31 episomes as well as in HPV positive biopsy tissue and cervical cancer cell lines containing integrated viral genomes [122,123,124]. Heterologous expression of IFN-κ in HPV positive keratinocytes induces an anti-viral state, with re-expression of numerous PRRs, IRFs, and ISGs, including STAT1 and IFIT1 that have been shown to block HPV replication [122]. Additionally, ectopic IFN-κ expression leads to an increase in Sp100, which localizes to HPV16 and HPV31 replication factories in keratinocytes and represses HPV31 gene expression and replication [125,126]. Furthermore, keratinocyte depletion of Sp100 using siRNAs results in an increase in HPV18 replication and transcription upon quasivirus infection, demonstrating that Sp100 acts as an intrinsic restriction factor for HPV infection [87].

Several mechanisms have been described for HPV-mediated repression of IFN-κ. The expression of the HPV16, 18, and 31 E6 and to a lesser extent E7 is sufficient to decrease expression of IFN-κ [122]. IFN-κ levels seem to mainly be regulated by E6 through epigenetic repression of the IFN-κ promoter by DNA methylation [123]. However, E2 expression is also associated with reduced levels of IFN-κ, although the mechanism of repression has not been determined [95]. In the context of the HPV16 genome, the loss of E5 leads to increased expression of IFN-κ as well as ISGs that correlate with an increased frequency of viral genome integration [124]. E5 represses IFN-κ expression by blocking TGF-β-induced signaling and stimulating epidermal growth factor receptor (EGFR)/mitogen activated protein kinase (MAPK) signaling [124]. Keratinocytes may express IFN-κ to maintain basal levels of ISGs such as Sp100 to suppress gene expression of incoming viruses. However, the expression of early genes allows HPV to repress IFN-κ expression, providing an environment supportive of viral replication and persistence.

## 7. Interference with IFN Signaling and ISG Production

Type I and Type III IFNs can act in an autocrine or paracrine manner to induce anti-viral activity through the expression of ISGs [127]. All Type I IFNs signal through a shared heterodimeric receptor, consisting of IFNAR1 and IFNAR2, whereas Type III IFNs signal through a heterodimeric receptor composed of IFNLR1 and IL10Rβ (Figure 3B). While the Type I receptor is expressed on all nucleated cells, the Type III IFN receptor is most abundant on epithelial cells [128,129]. Both Type I and Type III IFNs signal through the JAK/STAT pathway to trigger formation of the ISGF3 complex, which is composed of phosphorylated STAT1, STAT2, and IRF9 [127]. ISGF3 then translocates to the nucleus, where it binds to ISRE elements in the promoter region of ISGs, which encode proteins that generally function to inhibit viral replication [56].

As noted, numerous ISGs are reduced in expression in HPV infected cells, including STAT1, which is a critical component of the ISGF3 complex. Lack of STAT1 expression likely plays a critical role in HPV’s ability to repress ISG expression and promote viral replication. In support of this, ectopic expression of STAT1 results in loss of HPV31 episomes and the emergence of cells containing integrated viral genomes [61]. While several studies have shown that the overexpression of E6 and E7 alone as well as E2 is sufficient to decrease ISG levels, James et al. recently demonstrated that, in the context of the HPV16 genome, both E6 and E7 are required to synergistically repress ISG expression [130]. These studies were carried out using immortalized N/tert-1 keratinocytes that maintain wild-type HPV16 genomes or HPV16 genomes containing stop codons in the E6 or E7 open reading frame. Because E2 is expressed in the E6, E7 mutant-containing cells, how E2 contributes to repression of the IFN response in the context of infection is currently unclear. Overexpression studies have demonstrated that one mechanism by which E6 and E7 proteins repress ISG expression is through interference with the JAK/STAT signaling pathway. HPV18 E6 binds to and interferes with the activity of Tyk2, a member of the JAK family that promotes phosphorylation/activation of STAT1 and STAT2 upon IFN/receptor binding [131]. HPV16 E7 binds to IRF9, disrupting the activity of the ISGF3 complex in response to IFN-α [132,133]. Whether these interactions are conserved across high-risk E6 and E7 proteins, and if these interactions are important in suppressing IFN signaling in the context of infection, is currently unclear. However, HR HPV-infected cells are responsive to long-term treatment with IFN-β, resulting in episomal loss [64,65], suggesting that these interactions are not sufficient to completely block signaling through the JAK/STAT pathway.

## 8. Regulation of the IFN Response during the Productive Phase of the Life Cycle

Although several mechanisms have been identified by which HPV blocks production of IFN and/or interferes with ISG expression, the majority of these studies have been carried out in monolayer cultures and/or with overexpression of the HPV early genes. As such, our knowledge regarding the mechanisms that HPV employs to suppress an IFN response in differentiating keratinocytes is lacking. Several approaches are commonly used to study the differentiation-dependent phase of the viral life cycle. Organotypic raft cultures mimic in vivo epithelial differentiation and support all phases of the viral life cycle, including virion production [134]. Suspension in the semi-solid media methylcellulose as well as growth of cells in high calcium medium is sufficient to induce epithelial differentiation and activate the productive phase of the viral life cycle [134,135]. Methylcellulose- and calcium-induced differentiation have been the primary tools used to study how HPV regulates the innate immune response upon differentiation.

### 8.1. IFN Production and ISGs Impact Productive Viral Replication and Late Gene Expression

RNA-sequencing analysis of differentiating HPV16 positive NIKs as well as CIN1-derived HPV16 positive W12E cells revealed massive changes to the transcriptome upon calcium-induced differentiation, including the upregulation of genes associated with a Type I IFN response [136]. We and others have shown that Type I IFN-α/β as well as Type III IFN-λ1 increase in keratinocyte lines containing HPV16 or HPV31 genomes upon methylcellulose- or calcium-induced differentiation [115,137]. Treatment of CIN1-derived HPV31 positive CIN612 9E cells with recombinant IFN-α/β or IFN-λ1, 2, 3 induces ISG expression and blocks productive replication upon calcium-induced differentiation [115], demonstrating that IFN production must be minimized in order for late events in the viral life cycle to occur.

Several ISGs have been identified as restriction factors for late gene expression and/or productive replication. Depletion of IFI16, a dsDNA sensor as well as ISG, in NIK-HPV18 positive cells results in increased viral replication and transcription upon methylcellulose-induced differentiation through the removal of repressive heterochromatin on the early and late viral promoters [138,139]. Depletion of Sp100 in HPV31 positive CIN612 9E cells results in an increase in late gene expression and productive replication upon calcium-induced differentiation [125,126]. Furthermore, ISGylation of the L1 capsid protein by ISG15 has been shown to negatively impact virion production [140]. These results indicate that suppression of ISG expression is critical to completion of the viral life cycle. However, very little is known regarding the mechanisms by which HPV regulates an IFN response in differentiating keratinocytes and if these mechanisms differ from those characterized in undifferentiated cells. 

### 8.2. HPV Hijacks Apoptotic Caspase Activity to Regulate IFN Production upon Differentiation

**Figure 4:**
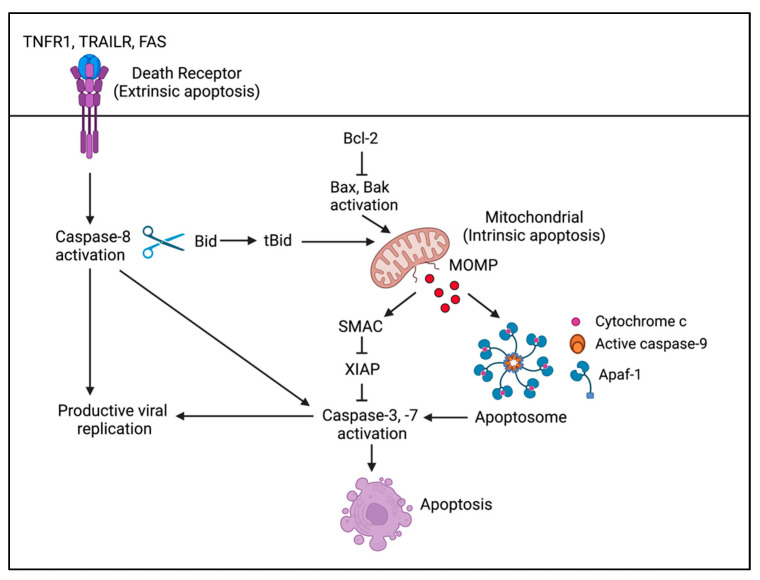
Overview of the extrinsic and intrinsic apoptotic pathways. The mitochondrial (intrinsic) pathway of apoptosis is activated by various cellular stresses (e.g., DNA damage, ER stress, ROS production, viral infection), resulting in mitochondrial outer membrane permeabilization (MOMP) and the release of cytochrome c, triggering formation of the apoptosome and activation of caspase-9. Caspase-9 then cleaves and activates the effector caspases, caspase-3 and -7, leading to apoptosis. The death receptor (extrinsic) pathway of apoptosis is stimulated by the binding of death receptor ligands (e.g., TNF, TRAIL, FASL) to their cognate death receptor and results in activation of the initiator caspase caspase-8. Under conditions of low levels of the inhibitor of apoptosis protein XIAP, which inhibits caspase-9, -3, -7, caspase-8 can directly cleave caspase-3. Under high XIAP conditions, caspase-8 must cleave the pro-apoptotic protein Bid to form tBid, which translocates to the mitochondria to stimulate MOMP by Bax/Bak. MOMP results in the release of SMAC, an inhibitor of XIAP, allowing for activation of caspases-9, -3, -7 [145]. Created with BioRender.com (accessed on 18 July 2022).

The mitochondria and apoptotic caspases (cysteine proteases) have been established as important regulators of innate immunity [141,142]. Apoptotic caspases were previously identified as having a pro-viral role in the productive phase of the HPV31 life cycle, with caspase-mediated cleavage of the E1 viral helicase being required for efficient productive replication [135]. Apoptosis is initiated by two converging pathways: intrinsic and extrinsic (Figure 4) [143]. In the intrinsic (mitochondrial) pathway, diverse apoptotic signals trigger mitochondrial outer membrane potential (MOMP) by the pro-apoptotic Bcl2 family members Bax and Bak, resulting in the release of cytochrome c, which binds to Apaf-1 and activates the initiator caspase, caspase-9. Caspase-9 then cleaves and activates the effector caspases, caspase-3 and -7 [142]. The extrinsic pathway is initiated by the binding of death receptor ligands to their cognate death receptor on the cell surface, resulting in the activation of the initiator caspase, caspase-8 [144]. Caspase-8 can directly cleave caspase-3 or initiate apoptosis through the intrinsic pathway by cleaving the pro-apoptotic Bcl2 family member Bid to tBid, which translocates to the mitochondria and stimulates MOMP by Bax/Bak [145].

**Figure 5:**
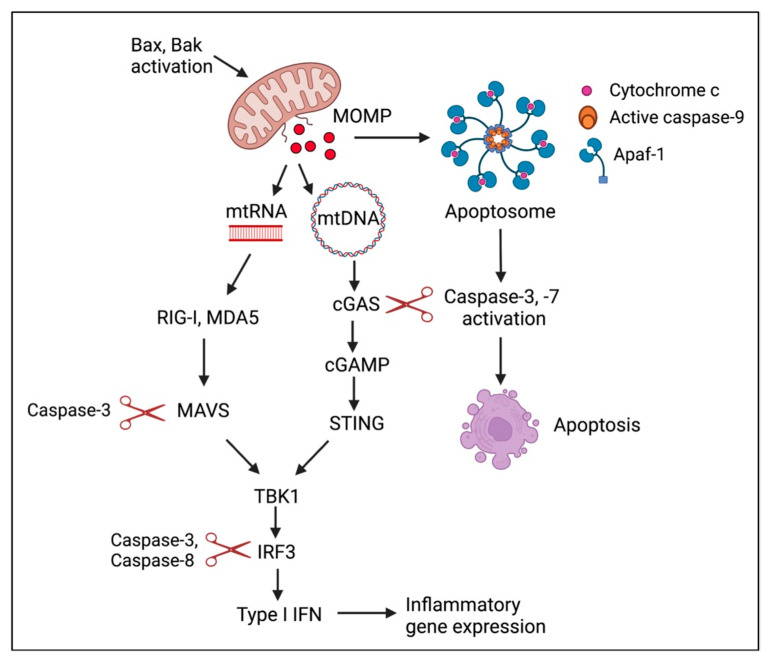
Apoptotic caspases block IFN production through cleavage of pathway components. Mitochondrial outer membrane permeabilization (MOMP) leads to the release of mtDNA as well as mtRNA that can be sensed by the cGAS/STING and MDA5/MAVS pathways, respectively, leading to Type I IFN production. Caspase activation resulting from MOMP blocks the IFN response by cleavage of various components of the cGAS/STING and RLR-MAVS signaling pathway. Activation of apoptotic caspase also suppresses IFN expression due to RNA and DNA virus infection (not shown). Created with BioRender.com (accessed on 18 July 2022).

MOMP triggers the release of mitochondrial DNA (mtDNA) that activates the cGAS-STING pathway, initiating a Type I IFN response that can result in a pro-inflammatory type of cell death (Figure 5) [146,147]. MOMP also leads to the release of mtRNA that can be sensed by the MDA5-MAVS pathway [148]. However, the concomitant activation of caspases downstream of MOMP attenuates this response to maintain apoptosis as an immunologically silent form of cell death [146,147]. Recent studies by Ning et al. demonstrated that caspase-3 cleaves cGAS, in turn inactivating sensing of cytosolic DNA (mtDNA as well as viral DNA) [149]. Caspase-3 also targets MAVS and IRF3 to regulate IFN production in response to mtRNA or RNA virus infection [149]. IRF3 is also a substrate of caspase-8 [150]. Caspase-8 can also block RIG-I signaling in response to RNA virus infection [151]. Apoptotic caspases can therefore regulate the IFN response to misplaced host nucleic acids as well as RNA and DNA virus infection [141,149]. Recent studies showed that KSHV uses caspase-8 activity to suppress an IFN response during lytic replication [152], raising the possibility that HPV may also use this non-death function of apoptotic caspases to regulate an IFN response during productive replication [115].

HPV31 activates low levels of apoptotic caspases (e.g., caspase-8, -9, -3, and -7) upon methylcellulose- and calcium-induced differentiation in the absence of morphological features of apoptosis and requires this activity for productive replication [135]. We found that CIN1-derived HPV16 positive W12E cells also exhibit apoptotic caspase activity upon differentiation [115]. Under conditions of caspase deficiency, calcium-induced differentiation of HPV31 and HPV16 positive keratinocytes results in a significant increase in the expression of Type I IFN-β and Type II IFN-λ1 as well as the expression of several ISGs, including IFIT1, ISG15, and OAS2 [115]. Additionally, the increase in IFN secretion is sufficient to block productive HPV31 replication in bystander cells. These results suggest that IFN production must be repressed upon differentiation to prevent autocrine/paracrine IFN signaling and ISG expression. Similar to KSHV, we found that HPV-induced caspase-8 activity plays a role in regulating the IFN response, but caspase-3 activity is also required [115]. Surprisingly, we found that the IFN response is not mediated by the cGAS-STING pathway, but instead occurs through the RNA sensing MDA5-MAVS-TBK1 pathway. Furthermore, knockdown of MDA5 or MAVS under caspase proficient conditions results in an increase in viral genome amplification upon differentiation, identifying MDA5 and MAVS as restriction factors for HPV31 [115]. The identification of the host and/or viral RNAs that are sensed by MDA5 as well as delineating the mechanism by which caspases thwart this response are important areas of future research. Overall, these studies demonstrate that HPV hijacks caspase activity to establish a replication-competent environment in differentiating keratinocytes at least in part by suppressing antiviral signaling. Additionally, these studies reveal a novel role for RNA in activating a cell intrinsic immune response during the productive phase of the HPV life cycle.

## 9. Summary and Future Directions

In this review, we discuss the multiple mechanisms that HPVs use to target the antiviral innate immune response. However, our knowledge regarding how HPV manipulates the cellular environment to avoid and/or block activation of the innate immune response during the course of the viral life cycle is just starting to be elucidated. Several outstanding questions remain: (i) how HPV’s ability to target components of the innate immune response contributes to long-term infection and cancer development, (ii) how HPV subverts activation of an antiviral response despite the presence of persistent DNA damage, which when mislocalized alerts cytoplasmic DNA sensors, and (iii) whether the mechanisms identified for HPV proteins to interfere with innate immune signaling in overexpression studies are conserved in the context of viral infection. Further understanding of how HPVs hijack cellular processes to subvert antiviral signaling may identify novel therapeutic targets for treatment of HPV-associated diseases.
